# ViralQC: a tool for assessing completeness and contamination of predicted viral contigs

**DOI:** 10.1093/bioinformatics/btag463

**Published:** 2026-08-21

**Authors:** Cheng Peng, Jiayu Shang, Jiaojiao Guan, Yanni Sun

**Affiliations:** Department of Electrical Engineering, City University of Hong Kong, Hong Kong (SAR), China; Department of Information Engineering, Chinese University of Hong Kong, Hong Kong (SAR), China; Department of Electrical Engineering, City University of Hong Kong, Hong Kong (SAR), China; Department of Electrical Engineering, City University of Hong Kong, Hong Kong (SAR), China

## Abstract

**Motivation:**

Viruses represent the most abundant biological entities on Earth, playing vital roles in diverse ecosystems. Cataloging viruses across various environments is essential for understanding their properties and functions. Metagenomic sequencing has emerged as the most comprehensive method for virus discovery. However, distinguishing viral sequences from the vast background of microbial organisms in metagenomic data remains a significant challenge. Existing tools experience varying degrees of false positive rates due to noise in sequencing and assembly, and the integration of proviruses into microbial genomes. This highlights the urgent need for an accurate and efficient method to evaluate the quality of viral contigs.

**Results:**

To address these challenges, we introduce ViralQC, a tool designed to assess the quality of viral contigs or bins. ViralQC identifies microbial contamination within putative viral sequences using an ensemble framework powered by DNA and protein foundation models and estimates completeness by analyzing protein organization. We evaluated ViralQC on multiple datasets and compared its performance against the state-of-the-art tool, CheckV. Leveraging both DNA and protein foundation models, ViralQC achieves higher sensitivity on contamination detection for contigs longer than 10 kbp while maintaining comparable accuracy. Additionally, ViralQC delivers more accurate estimation on contigs with completeness > 50%.

**Availability:**

The source code of ViralQC is available via: https://github.com/ChengPENG-wolf/ViralQC.

## 1 Introduction

Viruses are the most abundant biological entities on Earth, playing crucial roles in diverse ecological systems, from human habitats to extreme environments (Cobián Güemes *et al.* 2016). To better understand their properties and functions, it is essential to catalog viruses across various environments. Metagenomic sequencing has emerged as the most comprehensive approach for viral discovery, enabling the sequencing of all genetic materials, including viruses, from host-associated or environmental samples (Rahlff *et al.* 2023).

While metagenomic sequencing is powerful in studying viruses, the generated sequencing data are highly heterogeneous, with virus-originated reads often obscured by the overwhelming presence of reads from cellular organisms (Dolja and Koonin 2018). This makes the identification of virus-derived sequences within metagenomic data a significant challenge. To address this, several tools have been developed to identify viral contigs from assembled contigs. Alignment-based tools are efficient at identifying viruses based on sequence similarity, leveraging their comprehensive databases. Recently, learning-based components have been increasingly incorporated into virus identification workflows (Ren *et al.* 2020, Guo *et al.* 2021, Camargo *et al.* 2024, Peng *et al.* 2024). Although these learning-based tools differ in their training data, extracted features, and model architectures, they share the common goal of utilizing features beyond sequence similarity. For example, VirSorter2 (Guo *et al.* 2021) extracts 27 genetic features from sequences and integrates random forest classifiers to improve the detection of phages.

Despite the promising results of these virus identification tools, the quality of reported viral contigs remains a significant concern. Many of these contigs are fragmented and incomplete due to the inherent limitations in sequencing and assembly processes, resulting in the loss of critical genomic regions (Mallawaarachchi *et al.* 2023). Furthermore, contamination from host genomes is a prevalent issue, particularly for proviruses that integrate into microbial chromosomes (Nayfach *et al.* 2021b). When these integrated regions are assembled together with flanking microbial sequences, the resulting contigs contain non-viral segments that can confound downstream analyses such as functional annotation and evolutionary studies, ultimately distorting our understanding of viral communities. Thus, understanding the quality of predicted viral contigs, including both contamination regions and completeness, is crucial for providing valuable insights to users. For instance, a complete genome and its fragments may align to different taxa in the reference database (Roux *et al.* 2019). This inconsistency can lead to incorrect taxonomic classification results for metagenomic contigs. By applying a completeness cutoff, it is possible to reduce errors in taxonomic assignment for contigs.

Assessing the quality of viral contigs poses several challenges due to the unique and diverse characteristics of viral genomes. Unlike cellular genomes, viruses exhibit high diversity in their genetic architectures, lacking universally conserved single-copy marker genes that are typically used for completeness estimation (Parks *et al.* 2015). CheckV (Nayfach *et al.* 2021a) applies protein alignments to the reference database and computes average amino acid identities and alignment fractions to estimate the expected genome length.

Detecting microbial contamination in viral contigs introduces another layer of complexity. Existing approaches often rely on aligning sequences with reference databases. CheckV identifies virus-host boundaries based on the presence of viral and microbial marker genes. However, the limited coverage of current viral reference databases and the reliance on well-curated marker genes or sequence motifs can lead to errors in marking contamination regions in viral contigs from understudied ecosystems.

To address these limitations, we aim to develop an accurate and efficient quality-assessment tool that (1) detects microbial contamination within putative viral sequences and (2) provides quantitative estimates of viral contig completeness.

### 1.1 Overview

In this work, we present a novel method ViralQC for automatically assessing the quality of viral sequences assembled from metagenomic data. First, it identifies and reports the microbial contamination within viral contigs. To achieve this, ViralQC employs a multi-modal ensemble framework that integrates both nucleotide and protein information from the input sequences. This framework is further strengthened by foundation models pre-trained on 32 billion nucleotide bases and 138 million proteins, enabling accurate detection of non-viral regions. As these foundation models are large language models, the contamination detection module requires GPU resources. Second, ViralQC provides a quantitative estimate of viral contig completeness based on protein organization. While viruses mutate at high rates and evolve rapidly, blocks of conserved linear order of genes persist among related viral genomes, such as those infecting the same host (Seitzer *et al.* 2018). Based on the protein organization, ViralQC identifies the most closely related reference virus for each input contig and uses this to estimate how complete the contig is. For viruses with small genomes encoding only a few proteins, ViralQC leverages protein sequence similarity to support this assessment. As metagenomic sequencing continues to uncover novel viruses at a rapid pace, ViralQC also provides a script that enables users to construct a custom complete genome database from their own sequences, ensuring that the reference set remains up to date.

We evaluated ViralQC on multiple datasets, including a provirus dataset, a leave-one-taxon-out dataset, and metagenomic sequencing data. For the first two datasets with known labels, we ensured no overlap between the training and test data. To be specific, the leave-one-taxon-out experiment evaluated the model’s performance on a new taxon (e.g. a new species, genus, or family) that is not included in the training set. This experiment investigated ViralQC’s capacity to assess the quality of distantly related viruses. In addition, we benchmarked ViralQC against CheckV (Nayfach *et al.* 2021a), which is the state-of-the-art tool for virus quality quantification.

## 2 Methods

**Figure 1:**
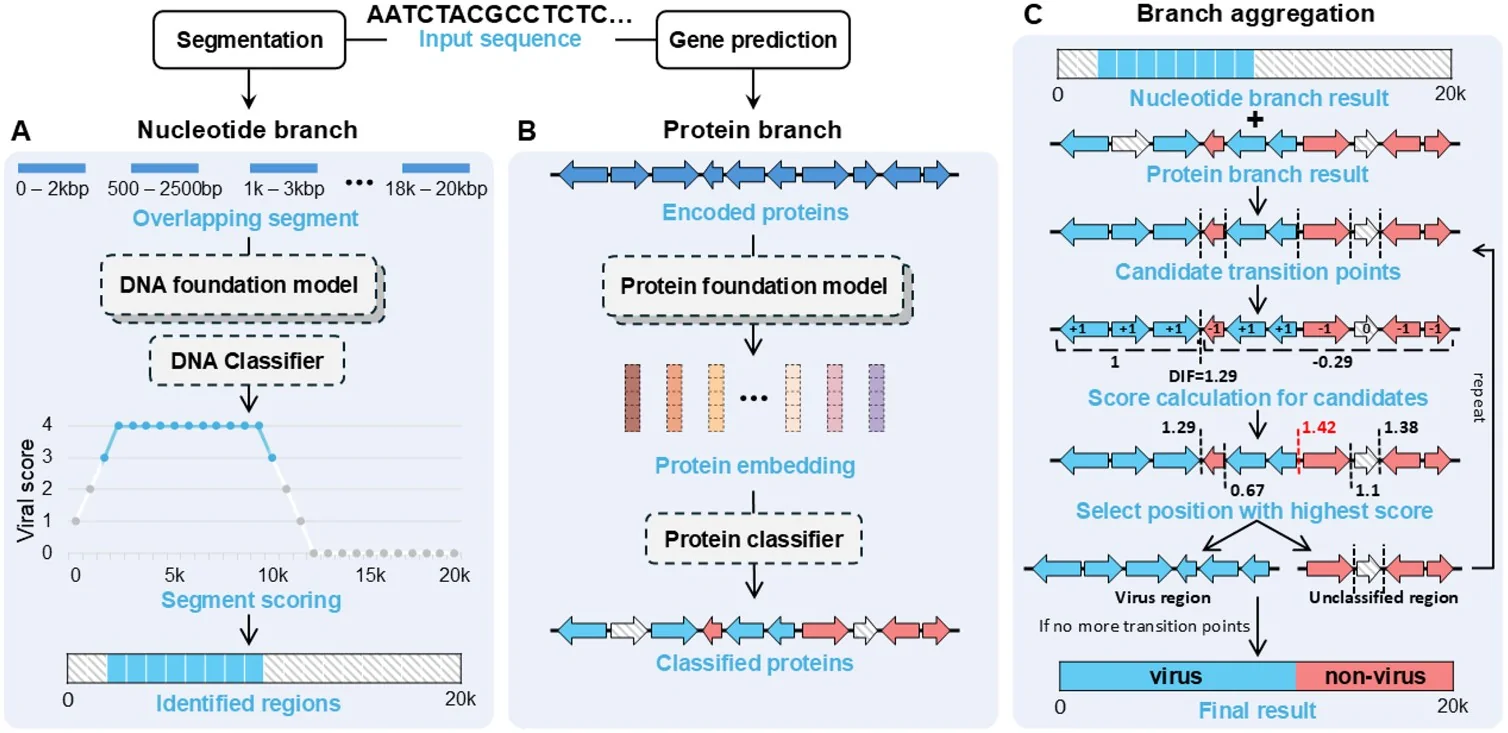
The framework of the contamination detection module. (A) The nucleotide branch identifies viral regions based on sequence-level features using a fine-tuned DNA foundation model. (B) The protein branch classifies whether an encoded protein is virus-originated. (C) The results from both branches are aggregated and refined to produce the final predictions of virus and non-virus regions. There are five candidate transition points (dashed lines). For each, ViralQC computes the mean score on its left and right sides and retains the boundary with the largest difference (DIF = 1.42). After calculating the average score on both sides of a candidate, the one with the highest difference is kept (DIF = 1.42). Then, the left segment is classified as viral and excluded from the next iteration. The DIF values of the remaining candidates in the unresolved region are recalculated. If no candidate meets the DIF threshold, the procedure stops.

**Figure 2:**
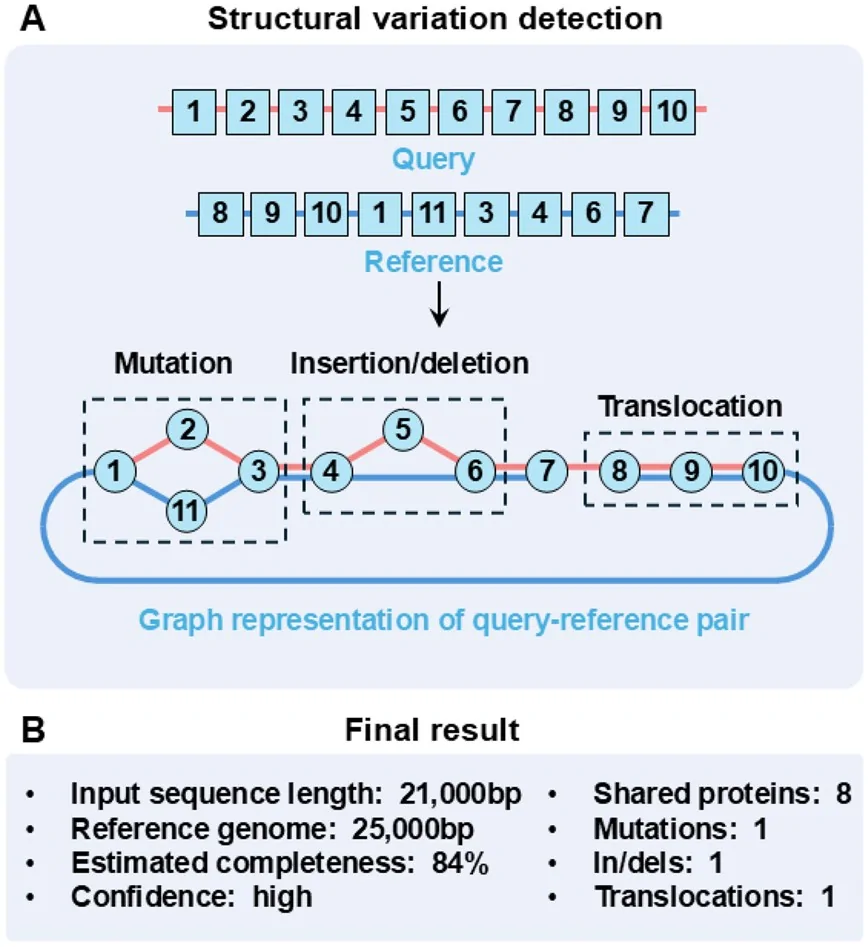
(A) The detection of SVs using a graph-based method. Each query-reference pair is converted to a graph and the SVs are detected by analyzing the topology of the graph. (B) The completeness of the input sequence is estimated and reported based on the most related reference genome.

ViralQC is organized into two primary modules. The contamination detection module (Fig. 1) identifies boundaries of non-viral regions in input contigs. The completeness estimation module (Fig. 2 and Fig. S1, available as supplementary data at *Bioinformatics* online) evaluates the expected genome length and calculates the completeness of the viral contigs. Below we provide a detailed description of the methodology in each module.

### 2.1 Contamination detection module

To identify potential contamination in viral contigs, ViralQC employs an ensemble framework that integrates nucleotide and protein features through two separate specialized branches. As noted by (Kim and Steinegger 2024), these two feature types offer complementary strengths: protein-based features are more sensitive for detecting remote homology relationships, while nucleotide-based features provide finer resolution for distinguishing between different taxonomic groups. By integrating both, ViralQC achieves more robust contamination detection than either alone. To further improve generalization to novel or underrepresented sequences, ViralQC incorporates DNA and protein foundation models, which are large language models pre-trained on extensive and diverse datasets. These models capture complex sequence patterns that traditional methods may overlook (Li *et al.* 2024).

Figure 1 sketches the main components of the contamination detection module. Given an input viral contig, ViralQC processes it through a nucleotide branch and a protein branch. The results provided by both branches are aggregated, including the start and end positions of identified contamination regions. Below we describe the details of each branch.

#### 2.1.1 Nucleotide branch

The nucleotide branch is designed to classify viral regions based on the nucleotide composition of sequences. We leverage a fine-tuned DNA foundation model, DNABERT-2 (Zhou *et al.* 2023), as the engine of the nucleotide branch to evaluate whether an input sequence is of viral origin. DNABERT-2 was pre-trained on genomes from 135 species spanning vertebrates to bacteria, enabling it to learn diverse and valuable representations of DNA sequences. To adapt the DNA foundation model for viral region detection, we fine-tuned it to a binary classification task with two labels (virus and non-virus) on viral and bacterial sequences (Supplementary 1.1, available as supplementary data at *Bioinformatics* online).

The workflow of the nucleotide branch is illustrated in Fig. 1A. First, each contig is divided into overlapping 2 kbp segments with a 500 bp sliding step, ensuring that each 500 bp region (except the ends of the contigs) is covered by four overlapping 2 kbp segments. The fine-tuned DNA foundation model is adopted to decide whether each segment is either viral or non-viral. If a 2 kbp segment is classified as a viral region, then it will be assigned a score of 1.

A viral score between 0 and 4 is assigned to each 500 bp region, based on the number of covering segments predicted as viral. Regions with higher viral scores indicate stronger viral signals. Consecutive regions with viral scores greater than 2 are merged and labeled as viral regions. This overlap-based scoring approach effectively reduces false positives generated by learning models and ensures accurate detection of viral regions.

#### 2.1.2 Protein branch

The protein branch focuses on the prediction and classification of viral or non-viral proteins encoded by the input (Fig. 1B). It is designed to complement the nucleotide analysis by providing a higher-level understanding of the sequence’s protein composition. This dual approach ensures that ambiguous or weak signals in the nucleotide branch are cross-validated with protein-based predictions.

To implement this, the protein branch utilizes the protein foundation model ESM2 (Lin *et al.* 2023), which is pre-trained on protein sequences from the UniRef database (Suzek *et al.* 2015, Devlin *et al.* 2018). For each protein, ESM2 generates a per-amino-acid embedding of $d_{l}\times d_{e}$, where $d_{l}$ is the number of amino acids in the protein sequence, and $d_{e}$ is the embedding size. The embedding is a meaningful representation of the protein sequence that encapsulates sequence composition in a high-dimensional space, making it suitable for classifying proteins.

The protein branch begins by predicting proteins from input contigs using Prodigal-gv (Camargo *et al.* 2024), a modified version of Prodigal (Hyatt *et al.* 2010) that optimizes gene calling for giant viruses and viruses that use alternative genetic codes. Then the predicted amino acid sequences are encoded into per-amino-acid embeddings using ESM2. Embeddings are obtained by averaging over all amino acids to reduce computational complexity. A binary classifier is trained to classify these proteins based on their embeddings (Supplementary 1.1, available as supplementary data at *Bioinformatics* online). The classifier takes a $d_{e}$-dimensional input and returns a score between 0 and 1, indicating the likelihood of the protein being a viral protein. Stringent thresholds are employed to eliminate false positives and false negatives. Specifically, proteins with scores above a threshold (0.5) are classified as viral proteins, while those below a threshold (0.1) are classified as *non-viral*. Proteins falling between these thresholds refer to ambiguous cases and are classified as *unknown*.

#### 2.1.3 Branch aggregation

After obtaining results from both the protein and nucleotide branches, ViralQC aggregates these findings to predict the contamination regions (Fig. 1C). First, the protein classification results are refined using the viral regions detected by the nucleotide branch. Specifically, if more than 50% of an unknown/non-viral protein’s coding region overlaps with a viral region identified by the nucleotide branch, it is reclassified as a viral protein. After this refinement, consecutive proteins with the same label are merged to form larger, unified regions.

However, small fragmented regions may occur when viral and non-viral regions are not clearly delineated, particularly in cases where genes with conflicting labels are interspersed across the contig. To remove false predictions and define clearer boundaries, we search for *transition points*-protein boundaries where the protein composition changes significantly (viral protein-abundant to non-viral protein abundant or vice versa). By definition, transition points can only occur at protein edges. Therefore, we limit candidates to boundaries between viral and non-viral proteins identified in the previous step to narrow the search space. Each protein is assigned a score based on its label: +1 for viral, 0 for unknown, and −1 for non-viral. ViralQC scans the candidates along the contig. For each candidate, the average scores of the left side (from the start of the contig to the candidate) and the right side (from the candidate to a position that includes at least 10% of the contigs’ genes) are calculated. A candidate is identified as a *transition point* if it yields the largest absolute score difference between the two sides among all candidates and this difference exceeds a threshold. We set the threshold for score difference between left and right regions to 1 to ensure significant gene content differences between the two sides. Once a *transition point* is found, the region to its left is classified as viral/non-viral based on its gene content and excluded from further consideration. We repeat this process on the remaining segment until no additional transition points are detected. This procedure delineates true viral–non-viral boundaries and prevents over-fragmentation, improving ViralQC’s robustness on complex contigs.

### 2.2 Completeness estimation module

ViralQC estimates the completeness of an input viral contig in two steps. First, it identifies the most closely related reference genome based on protein organization similarities (Fig. S1B, available as supplementary data at *Bioinformatics* online). Notably, the reference database contains only complete viral genomes. Then, the completeness is calculated as the ratio of the input contig length to the length of the identified reference genome, which is a value ranging from 0 to 1 (Fig. 2B). In addition, ViralQC detects Direct Terminal Repeats as evidence for a complete genome (Supplementary 1.2, available as supplementary data at *Bioinformatics* online).

Beyond single contigs, ViralQC also supports completeness estimation for entire bins using the “–bin” option. This feature facilitates the assessment of the completeness of bins and segmented viruses. In single-contig mode, each input sequence is treated as a separate virus. In bin mode, all sequences within a bin are first concatenated into a single composite sequence. ViralQC then estimate the completeness following the same procedure as in single-contig mode.

#### 2.2.1 Identifying the closest reference by protein organization

To accurately estimate the completeness of a viral contig, it is essential to identify the most closely related reference genome. Traditional approaches often rely solely on sequence similarity. However, in real cases, most of the viral contigs lack close homologs in the reference database. As a result, sequence similarity is insufficient for highly divergent or novel viruses. To overcome these limitations, ViralQC incorporates not only sequence similarity but also the structural organization of proteins.

##### 2.2.1.1 Protein cluster

To incorporate both sequence similarity and protein organization, we construct protein clusters using complete viral genomes from NCBI RefSeq. First, protein sequences predicted by Prodigal-gv are clustered into protein clusters (PCs) at 50% identity and 80% coverage using MMseqs2 (Steinegger and Söding 2017). Proteins within the same cluster are considered homologous and perform similar biological functions. Each cluster is assigned a unique PC ID. With proteins labeled by their corresponding PC ID, each genome is represented as a series of protein clusters, indicating both the protein composition and the order of proteins within the genome. Examples of such digital representations of protein organization are illustrated in Fig. S1A, available as supplementary data at *Bioinformatics* online.

##### 2.2.1.2 Structural variation

To assess organization similarities at the protein level, ViralQC identifies four types of structural variations (SVs) between two sequences: mutations, insertions/deletions, translocations, and duplications. Mutation refers to an alteration of a gene. Insertion involves the acquisition of external genes from other viruses or hosts, while deletion is the loss of genes. Translocation refer to the rearrangement of DNA fragments within the genome that changes genes’ positions. Duplication changes gene copy numbers and is a key evolutionary mechanism driving genome expansion in giant viruses (Machado *et al.* 2023). These events induce differences in gene organization, which are considered to reduce protein organization similarity. Fewer SVs suggest that two sequences share a more similar gene organization and are therefore more evolutionarily related.

##### 2.2.1.3 Structural variation detection using graph algorithms

Given an input viral contig, ViralQC first conducts gene prediction and translation using Prodigal-gv. The predicted proteins are aligned to the proteins in the complete viral genome database using DIAMOND v2.1.9 (Buchfink *et al.* 2021). The “ultra-sensitive” option is specified to enhance the detection of distantly homologous proteins while maintaining computational efficiency. Based on the alignment results, the query proteins are labeled by the PC IDs of their best hits. Proteins without significant hits are treated as singletons and assigned unique PC IDs. Then the input contig is represented by a series of proteins in the same way as the complete genomes in the database.

ViralQC utilizes a graph-based method to detect SVs between the query and reference genomes. In this approach, both sequences are converted into a graph where nodes represent proteins in the sequences and an edge exists between two proteins if they are adjacent in any of the sequences. Proteins shared between the query and reference (with the same PC ID) are represented by the same node. Since query contigs are often incomplete and align to only part of a reference genome, ViralQC focuses on the aligned region rather than the entire genome. Proteins in the reference genome outside the aligned region are excluded, while query proteins outside the aligned region are penalized during scoring.

To detect SVs, ViralQC analyzes the topology of the constructed graph. Duplications can be easily identified by examining the number of proteins with identical PC IDs within the same sequence. Sequence-specific nodes that are present in only one of the two sequences may arise from mutations, insertions, or deletions. Specifically, ViralQC scans the whole graph and records all unique nodes and their neighbors. Then, mutations are defined as pairs of unique nodes (or consecutive unique nodes for multiple mutations) that share the same neighbors. Insertions refer to nodes that appear only in the query contig, while deletions are nodes unique to the reference genome. Translocation events are more difficult to estimate directly. Instead, ViralQC divides the graph into subgraphs (synteny blocks) by iteratively eliminating edges whose two end proteins are not adjacent in both sequences. Proteins within a synteny block are conserved in order, but the order of synteny blocks themselves may vary. The number of synteny blocks acts as an indicator of translocations. Figure 2A illustrates an example of these SVs displayed in the genome graph.

To determine the most closely related reference genome, ViralQC calculates a protein organization similarity score that incorporates both shared proteins and observed structural variations. The underlying assumption is that closely related reference genomes will share a higher number of proteins while exhibiting fewer structural variations. As a result, the protein organization similarity score is computed as the weighted sum of two components: the number of shared proteins and the number of observed SVs. Shared proteins contribute positively to the score, while SVs are penalized to account for deviations from the expected structural arrangement. In bin mode, however, insertions, deletions, and translocations are excluded from this calculation, as the ordering of sequences within a bin is unknown. The reference with the highest protein organization similarity score is selected as the most related genome and used to compute the completeness. In cases where multiple references achieve the same similarity score, or when the input contig shares few genes, the protein organization similarity approach may not be effective. In such cases, ViralQC selects the reference genome with the highest overall protein alignment score, calculated as the sum of the bit-scores of aligned proteins.

#### 2.2.2 Confidence level of completeness estimation results

While protein organization-incorporating homology search effectively identifies evolutionarily related genomes, false positives can occur when the query viral contigs are highly divergent from the database (e.g. share only a few proteins with the best hit). To address this, ViralQC reports a confidence level for each result, based on the number of shared proteins and genome size. Inspired by vConTACT (Bolduc *et al.* 2017), we assume that all *n* protein clusters have an equal probability of being chosen. The probability that two genomes with *a* and *b* protein clusters, respectively, share at least *c* clusters by chance is computed in Equation. 1. Then Equation 2 calculates the statistical significance score of query-reference pairs with *c* shared proteins out of the database size *T*. A significance score of 1 refers to an e-value of 1e-1, and a significance score of 5 refers to an e-value of 1e-5. Based on the significance score, ViralQC classify the results into three confidence levels: high confidence (score ≥ 5), medium confidence (1 ≤ score <5), and low confidence (score < 1).


(1) $$P(x\geq c)=\sum_{i=c}^{\min(a,b)}\frac{C_{a}^{i}C_{n-a}^{b-i}}{C_{n}^{b}}$$

(2) Confidence(A,B)=−log(P×T)

## 3 Result

We rigorously evaluated ViralQC on multiple datasets, summarized in Table S1 and detailed in Supplementary 1.3, available as supplementary data at *Bioinformatics* online, using the metrics outlined in Supplementary 1.4, available as supplementary data at *Bioinformatics* online. First, we assessed the contamination detection module on a provirus dataset, followed by benchmarking of the completeness estimation module on a leave-one-taxon-out dataset. We then applied ViralQC to a lake metagenome to demonstrate its utility on real-world data (Supplementary 2.3, available as supplementary data at *Bioinformatics* online). Finally, we measured the running time and peak memory usage during execution (Supplementary 2.4, available as supplementary data at *Bioinformatics* online).

### 3.1 Contamination detection

#### 3.1.1 Performance on provirus dataset

We evaluate ViralQC’s contamination detection performance on the provirus dataset, which contains 50 bacterial genomes with 241 manually annotated proviruses. Our experiments show that the viral contigs are often fragmented with incomplete genes at both ends and contain non-viral regions of different lengths (Fig. S2 and S6, available as supplementary data at *Bioinformatics* online). Correspondingly, we simulate contigs of varying lengths (5–50 kbp) and contamination levels (10%–50%) while preserving realistic host-virus junctions from the provirus sequences. To assess the impact of different features, we establish an ablation study by examining ViralQC’s performance using only DNA or protein features for contamination detection. As shown in Fig. S4A, available as supplementary data at *Bioinformatics* online, the model utilizing protein features outperforms the one using DNA features. This is likely because protein sequences, composed of a larger alphabet (25 amino acids), provide richer information compared to DNA sequences. While the model using protein features alone performs well, combining both DNA and protein features significantly enhances performance, emphasizing the importance of multi-modal feature integration.

**Figure 3:**
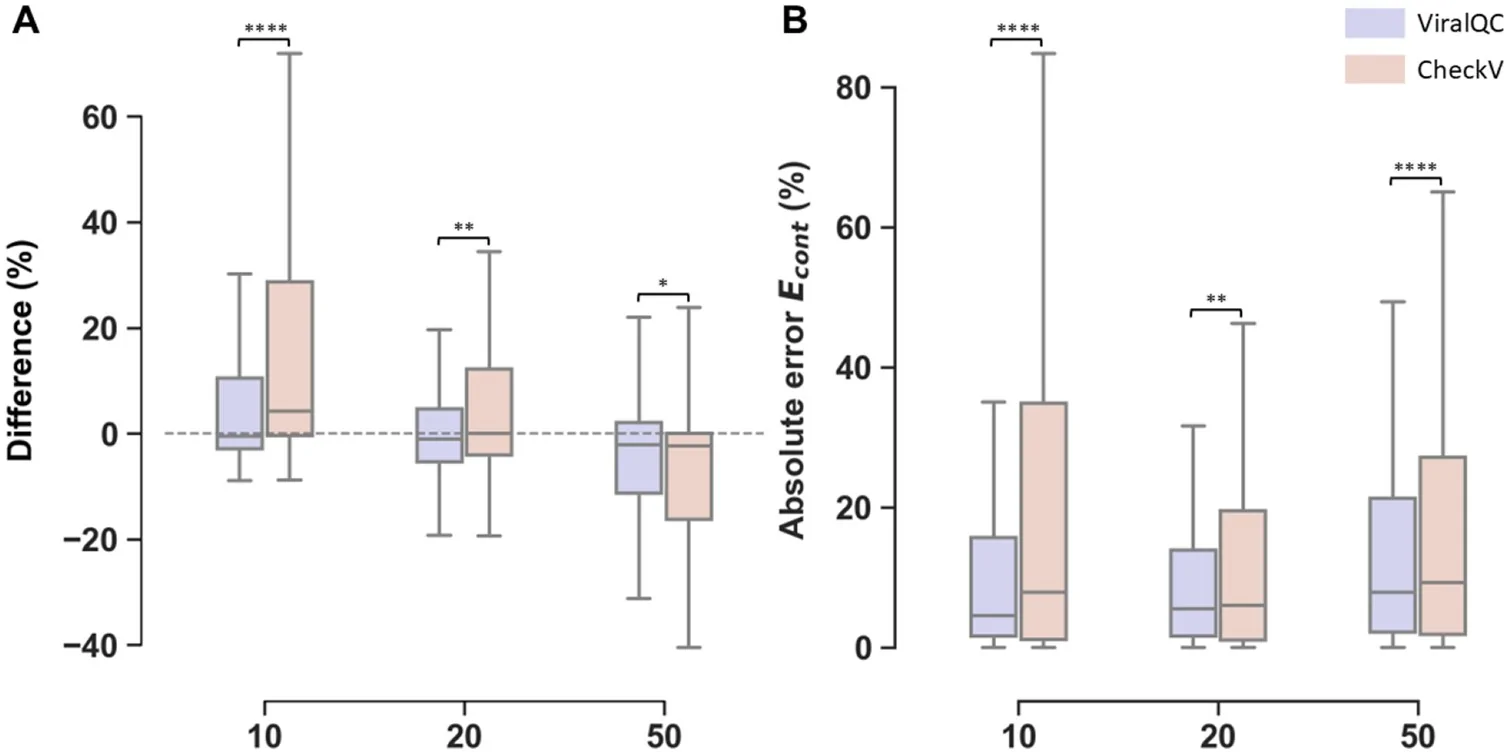
Evaluation of contamination detection on the provirus dataset. Each box summarizes the distribution across all provirus contigs detected by the respective tool at that contamination level. Contigs of all simulated lengths (5–50 kbp) are pooled together. (A) Signed difference between estimated and true contamination percentages across contigs with varying contamination levels (10%, 20%, 50%). Values closer to zero indicate more accurate contamination boundaries; positive values indicate overestimation; negative values indicate underestimation. (B) Distribution of absolute errors at varying contamination levels. Lower value is better.

Next, we compare ViralQC’s performance with CheckV (v1.0.3). As shown in Fig. S4B, ViralQC identifies a higher proportion of proviruses on contigs longer than 10 kbp across all contamination levels. Overall, ViralQC is able to detect 86% of provirus sequences, significantly outperforming CheckV, which identified only 62%. This improvement stems from ViralQC’s use of both nucleotide and protein information, whereas CheckV relies solely on marker genes to identify non-viral regions, highlighting ViralQC’s superior sensitivity in detecting host contamination within long provirus sequences. We also record the difference of contaminations estimated by each tool against the true contamination in Fig. 3A and the distribution of absolute errors in Fig. 3B. Both tools overestimate on contigs with low contamination (10%–20%) and tend to underestimate on contigs with high contamination (50%). ViralQC achieves a median error of approximately 6%, comparable to CheckV’s 8% but with lower variability, demonstrating robust performance across contamination levels.

We also evaluate the performance of both tools on 2382 contigs of purely viral origin. Figure S4C, available as supplementary data at *Bioinformatics* online shows that ViralQC has a minimal tendency to misclassify purely viral sequences as contaminated, with a false positive rate as low as 1%. This high specificity further solidifies ViralQC as a robust and reliable solution for identifying contamination. Although the inputs for ViralQC are supposed to be viral contigs, the input viral contigs generated by virus identification tools may contain false positives (bacterial contigs) in experiments. Thus, we also conducted an evaluation on 1744 contigs of purely bacterial origin in Fig. S4D, available as supplementary data at *Bioinformatics* online. ViralQC can identify most of the bacterial contigs, while CheckV considers these contigs to be viral contigs. The ability to detect potential bacterial contigs enables ViralQC to be a useful tool in practical use.

### 3.2 Completeness estimation

Because completeness is estimated by comparing protein organization against a reference set of complete viral genomes, the evaluation design requires careful consideration. To prevent data leakage, we ensure that test sequences are separated from the reference set and assess performance across varying levels of similarity to the reference genomes. Furthermore, since ViralQC provides a script that allows users to build a custom reference database, we benchmark ViralQC and CheckV (v1.0.3) using the same reference database to ensure a fair comparison of the underlying algorithms. Specifically, we consider two reference sets: one constructed from RefSeq, and one from CheckV. Under both settings, ViralQC delivers more accurate estimates than CheckV on contigs with completeness > 50%.

#### 3.2.1 Performance on leave-one-taxon-out dataset

**Figure 4:**
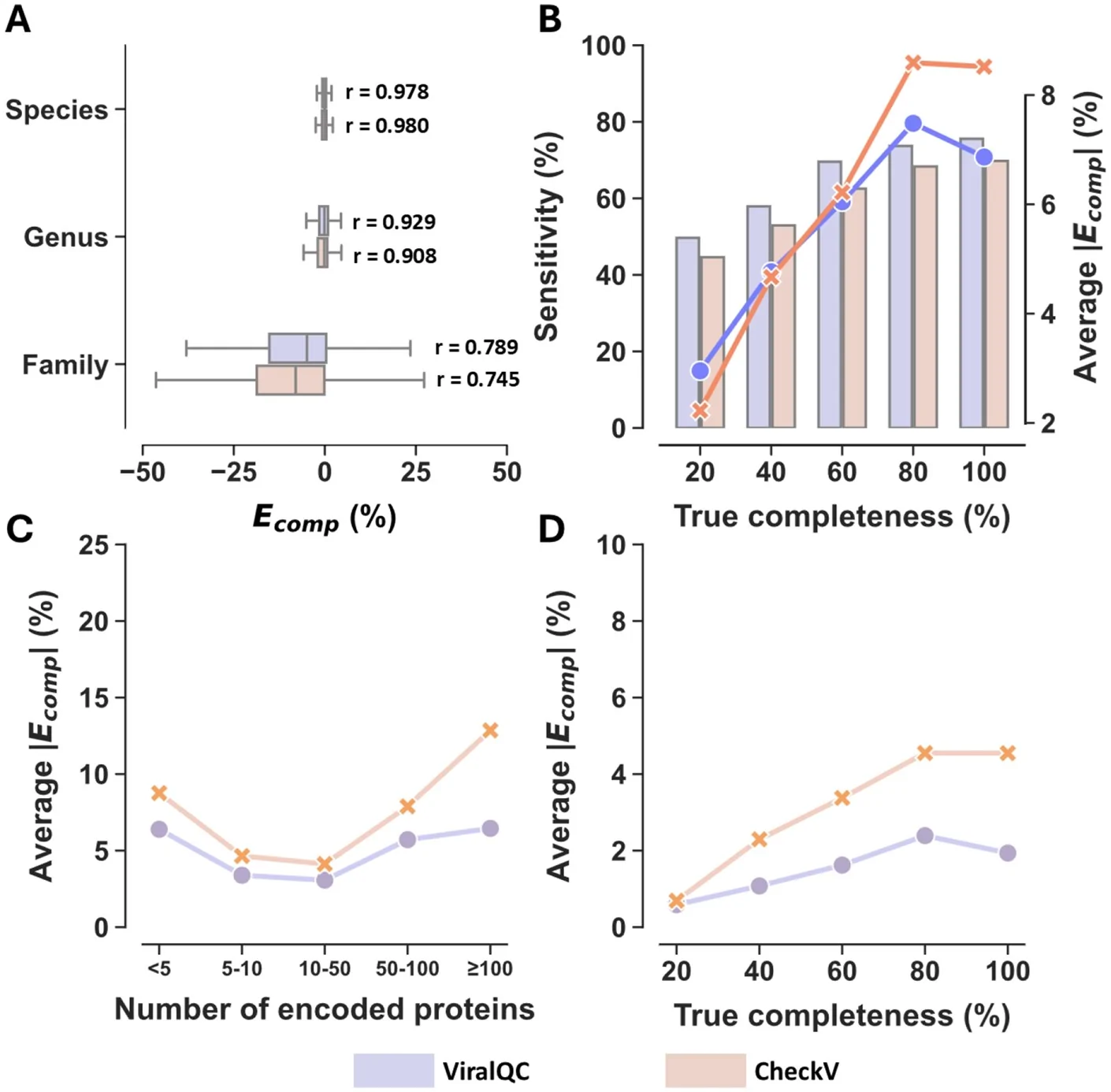
Evaluation of completeness estimation on the leave-one-taxon-out dataset. (A) Distribution of estimation errors at different taxonomic levels. Values closer to zero indicate better estimation; positive values indicate overestimation; negative values indicate underestimation. Higher Pearson’s correlation coefficient values (*r*) indicate stronger correlation. (B) Bars: the percentage of contigs with completeness estimates produced by CheckV and ViralQC at varying completeness levels; Lines: the corresponding average absolute errors. Lower values indicate better accuracy. (C) Completeness estimation performance grouped by the number of encoded proteins (<5, 5–10, 10–50, 50–100, ≥100). Lower values indicate better accuracy. (D) The average absolute error for both tools using the CheckV v1.5 database. Lower values indicate better accuracy.

We present the performance of completeness estimation using leave-one-taxon-out datasets, which simulate increasingly challenging scenarios by excluding certain taxonomic groups from the reference set. We first characterized the genome-size distribution of the dataset (Fig. S3, available as supplementary data at *Bioinformatics* online), which spans a broad range from 465 bp to 1.91 Mbp, with approximately 56% of genomes shorter than 5 kbp (encoding fewer than 5 proteins). This distribution confirms that both small and large viral genomes are adequately represented in our evaluation. As shown in Fig. 4A, the average absolute error increases from 1.9% at the species level to 13.1% at the family level. This pattern reflects the greater genomic diversity observed at higher taxonomic levels, especially at the family level. Despite the challenge carried by the leave-family-out dataset, both ViralQC and CheckV achieve high performance at the species and genus levels, with Pearson’s correlation coefficient (r) exceeding 0.9, indicating a strong relationship between the estimated and true completeness values.

We further compare their performance on contigs with varying completeness levels, ranging from 20% to 100%. As shown in Fig. 4B, ViralQC is able to estimate completeness for a large proportion of contigs over all ranges. Although CheckV performs better on short fragments with completeness <50%, ViralQC produces more accurate results for medium- to high-quality (>50% completeness) contigs. The results highlight ViralQC’s ability to effectively utilize the informative feature of protein organization for analyzing complete or near-complete contigs. This is particularly important in metagenomic studies, where high-quality contigs are often prioritized for downstream analyses. We also show the completeness estimation performance grouped by the number of encoded proteins in Fig. 4C. Errors decline for both tools as the protein count increases from 5 to 50, but rise again for contigs encoding more than 100 proteins. A likely cause is greater genome-size variability among large viruses, which makes completeness estimation less precise. Notably, ViralQC’s performance remains relatively consistent across all protein-count groups, with an average absolute error of 6% on contigs encoding fewer than 5 proteins and 3% on those encoding 10–50 proteins.

A dedicated benchmark further demonstrates that ViralQC’s bin mode—in which multiple contigs are jointly treated as a single genomic unit for completeness estimation—produces estimates comparable to those obtained under single-contig mode on the same genomes, despite the absence of gene-order information (Supplementary 2.2, available as supplementary data at *Bioinformatics* online), supporting its applicability to viral bins.

#### 3.2.2 Performance of ViralQC with CheckV’s reference database

Given the limited representation of environmental viruses in the NCBI RefSeq database, CheckV provides a more comprehensive database that includes sequences from metagenomes. This database is substantially larger than the one used in our earlier experiments, containing 62 895 sequences compared to 17 007. To assess the impact of this expanded reference set, we evaluated both ViralQC and CheckV on the leave-one-taxon-out dataset using the CheckV v1.5 database. As shown in Fig. 4D, the absolute error reduced substantially for both tools compared to the earlier results, highlighting the importance of a comprehensive reference database for capturing the high diversity of the virome. To this end, we provide a combined reference database integrating the CheckV database with the latest RefSeq complete genomes, available on our GitHub repository. Additionally, we include a script that allows users to expand this database with their own sequences, ensuring the reference set can grow alongside the rapidly expanding catalogue of known viruses.

#### 3.2.3 High-confidence predictions yield reliable results

**Figure 5:**
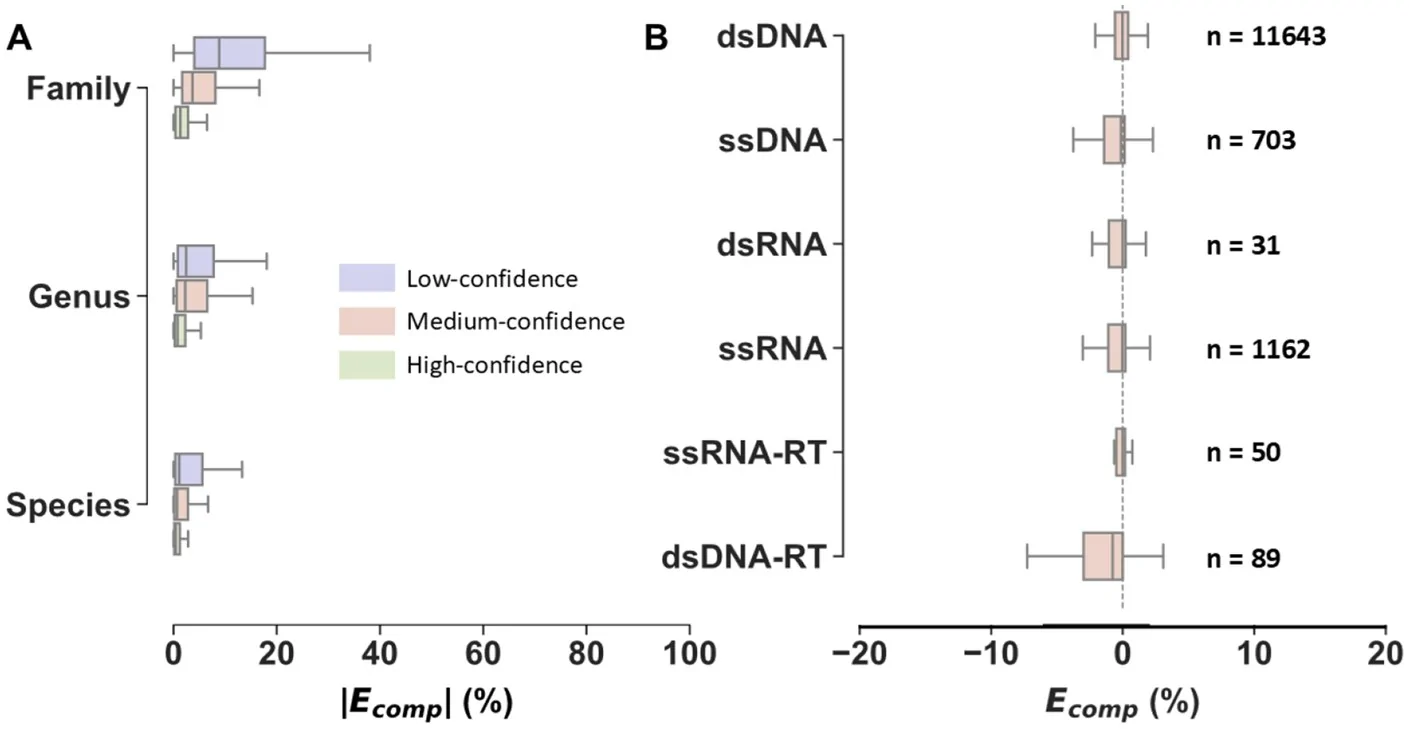
(A) ViralQC’s performance at different taxonomic levels grouped by confidence levels (low-, medium-, and high-confidence). The confidences are computed based on the number of shared proteins and genome size. Lower error indicates better accuracy. (B) High-confidence results of ViralQC on viruses based on their Baltimore classification. Values closer to zero indicate better estimation; positive values indicate overestimation; negative values indicate underestimation.

Although estimation accuracy degrades under the leave-family-out setting, we find that ViralQC’s reported confidence level serves as an effective indicator for identifying reliable predictions even in this challenging case. As shown in Fig. 5A, the average absolute error at the family level decreases substantially from 13% for low-confidence predictions to 3% for high-confidence predictions. This indicates that high-confidence estimates remain accurate even when the query virus has no close references in the database. We therefore recommend that users pay close attention to the confidence levels reported by ViralQC  prioritizing high-confidence estimates and cautiously interpreting low-confidence predictions when analyzing viruses from metagenomic data.

We further investigate ViralQC’s behavior across the six Baltimore classes using high-confidence predictions in Fig. 5B. Across all classes, the average error remains within a narrow range of −0.02% to −1.57%, demonstrating that ViralQC produces near-unbiased completeness estimates for the majority of viral lineages. dsDNA viruses, which dominate the reference database (*n* = 11 643), show the tightest error distribution centered at zero (average error −0.02%), reflecting the abundant complete genomes available for protein-organization-based reference matching in this class. ssRNA, dsRNA, ssDNA, and ssRNA-RT viruses exhibit a small but consistent negative error (average error between −0.32% and −1.44%). This pattern is intrinsic to ViralQC’s completeness estimation algorithm: when building the graph, ViralQC focuses on the aligned region rather than the entire genome, and proteins in the reference genome outside this region are excluded. Consequently, a larger reference can preserve or increase the number of shared proteins without penalty, creating an implicit preference for larger references during best-hit selection.

The largest deviations are observed for dsDNA-RT viruses, which show the widest error distribution (interquartile range spanning 10%). This class includes *Caulimoviridae* and *Hepadnaviridae*, which possess compact genomes (3–8 kbp) with extensively overlapping open reading frames (Lauber *et al.* 2017). The dense ORF overlap reduces the number of independently informative proteins, weakening the protein-organization signal on which ViralQC relies for reference selection. The limited number of dsDNA-RT references in the database (*n* = 89) further amplifies this effect by reducing the chance of finding a size-matched best hit. Despite these challenges, the average error for dsDNA-RT remains below 1.6%, confirming that ViralQC delivers reliable, lineage-agnostic completeness estimation across all Baltimore classes.

## 4 Discussion

In this work, we propose ViralQC, a tool designed for the quality assessment of metagenome-assembled viral contigs. ViralQC has two primary functions: detecting contamination within viral contigs and estimating their completeness. The major advancement in the contamination detection module stems from the adoption of foundation models. By leveraging both DNA and protein features, ViralQC can accurately identify virus-non-virus boundaries, enabling precise detection of non-viral regions. For completeness estimation, we implemented a novel graph-based approach that identifies the most related reference genome by analyzing protein organization.

Benchmarks on a provirus dataset demonstrate ViralQC’s high sensitivity and specificity in contamination detection. Notably, ViralQC detects more contamination on contigs longer than 10kbp with comparable accuracy and lower variability, whereas CheckV performs better on shorter contigs. For completeness estimation on a leave-one-taxon-out dataset, the performance of both ViralQC and CheckV drops substantially at the family level, reflecting the considerable genomic diversity among viruses from different families and beyond, which poses a fundamental challenge to completeness estimation. Despite this difficulty, ViralQC’s performance remains relatively consistent across a wide range of contig sizes, from short contigs with fewer than 5 proteins to those encoding more than 100 proteins. Specifically, ViralQC delivers more accurate estimates for medium- to high-quality (>50% completeness) contigs. To further explore whether the difference in completeness estimation is caused by the database or method, we also replace the reference database of ViralQC with the database of CheckV, which is roughly three times larger. With this expanded database, the error rates of both tools significantly improve. In particular, ViralQC’s overall error remains consistently lower than CheckV’s.

Beyond environmental applications, accurate quality assessment of viral contigs is particularly important for clinical metagenomics, where viral diagnostics increasingly rely on shotgun sequencing of patient samples. In such settings, fragmented or contaminated contigs can lead to false-positive pathogen calls or incorrect taxonomic assignments that confound clinical interpretation. By flagging contamination and providing confidence-stratified completeness estimates, ViralQC can help clinicians and clinical microbiologists prioritize high-confidence viral signals from complex backgrounds, supporting more reliable downstream surveillance and outbreak investigation.

While ViralQC significantly enhances the quality assessment of viral sequences, we see several avenues for optimization. One practical consideration is that the contamination detection module requires GPU resources due to the use of large foundation models. While this may increase the computational barrier for some users, the growing availability of GPU resources in academic and cloud computing environments makes this increasingly accessible. In the future, we plan to explore model distillation and quantization techniques to develop a lightweight version of the contamination detection module that can run efficiently on CPU-only environments.

## Supplementary Material

btag463_Supplementary_Data

## Data Availability

The data underlying this article are publicly available with their sources described in the article. The source code of ViralQC is available via: https://github.com/ChengPENG-wolf/ViralQC.
