# Supplementary material for: ViralQC: a tool for assessing completeness and contamination of predicted viral contigs
Source: Bioinformatics. 2026 Aug 21;42(Suppl 2):btag463. doi: 10.1093/bioinformatics/btag463 (PMC13501303; doi:10.1093/bioinformatics/btag463)
Supplement: Supplementary file 1 [file btag463_supplementary_data.pdf]

1 Supplementary for Methods and Materials

1.1 Contamination detection module

1.1.1 Training and Fine-tuning Details

Nucleotide branch We fine-tuned DNABERT-2 starting from its publicly available pre-trained checkpoint. A single linear classification layer was appended to the end of the model that serves as a binary classifier for classifying input sequences as viruses or non-viruses. The model was fine-tuned on the training dataset described in Table S1, which includes 49,929 viral genomes as positive samples and 1,651 bacterial/archaeal assemblies as negative samples, all fragmented into 200–3,000 bp segments. Training was performed on four NVIDIA GeForce RTX 4090 GPUs with the following hyperparameters: AdamW optimizer, learning rate 1×10 with 50 warm-up steps, and binary cross-entropy loss. It is important to note that although DNABERT-2 was not originally trained on viral sequences, its pre-training on 135 diverse species provides generalizable representations of nucleotide composition patterns. Fine-tuning on viral and bacterial sequences allows the model to learn discriminative features specific to this binary classification task.

Protein branch The ESM2 model was used as a fixed feature extractor with its weights frozen. Per-amino-acid embeddings generated by ESM2 were averaged across all positions to obtain a single protein-level representation. A two-layer feedforward classifier was then trained on these representations to distinguish viral from non-viral proteins, using the training data described in Section 1.2, with the following hyperparameters: AdamW optimizer, learning rate 1×105, and binary cross-entropy loss.

1.2 Completeness estimation module

1.2.1 Prediction of complete genomes

In addition to protein organization-based completeness estimation, ViralQC predicts complete genomes by detecting the presence of Direct Terminal Repeats (DTRs). DTRs are DNA sequences, typically several hundred base pairs long, that are repeated in the same orientation at both ends of a genome. They usually arise during assembly of short reads derived from circular genomes (Tisza *et al.*, 2020), or from linear genomes that can circularize after infection (Merrill *et al.*, 2016). As such, DTRs serve as an important marker for identifying complete genomes. Following the well-established algorithm in previous studies (Fernández-Pato *et al.*, 2025), ViralQC examines repeat regions at both ends of the sequence to detect DTRs. By incorporating DTR detection, ViralQC enhances its ability to accurately identify complete viral genomes, thereby improving the overall quality and reliability of metagenomic viral analyses.

1.3 Datasets

Training set for contamination detection To construct the dataset for training the models in nucleotide and protein branches of the contamination detection module, we downloaded viral genomes from the NCBI RefSeq database (Release 218). The genomes in the NCBI RefSeq database are manually curated and well-annotated, providing a reliable source of complete viral genomes. We also included a set of high-quality metagenomic viral sequences from previous studies (Guo *et al.*, 2021). These datasets include genomes from both known and novel viruses, resulting in a total of 49,929 viral genomes, which were used as positive training samples. To construct negative samples, we included microbial assemblies (bacteria and archaea) from NCBI RefSeq, the main sources of contamination in metagenomes. Since viral genomes are typically much shorter than microbial genomes, including all available microbial assemblies would introduce a large imbalance in cumulative sequence length between the two classes. To mitigate this, we down-sampled the negative dataset by randomly selecting two genomes per genus, yielding

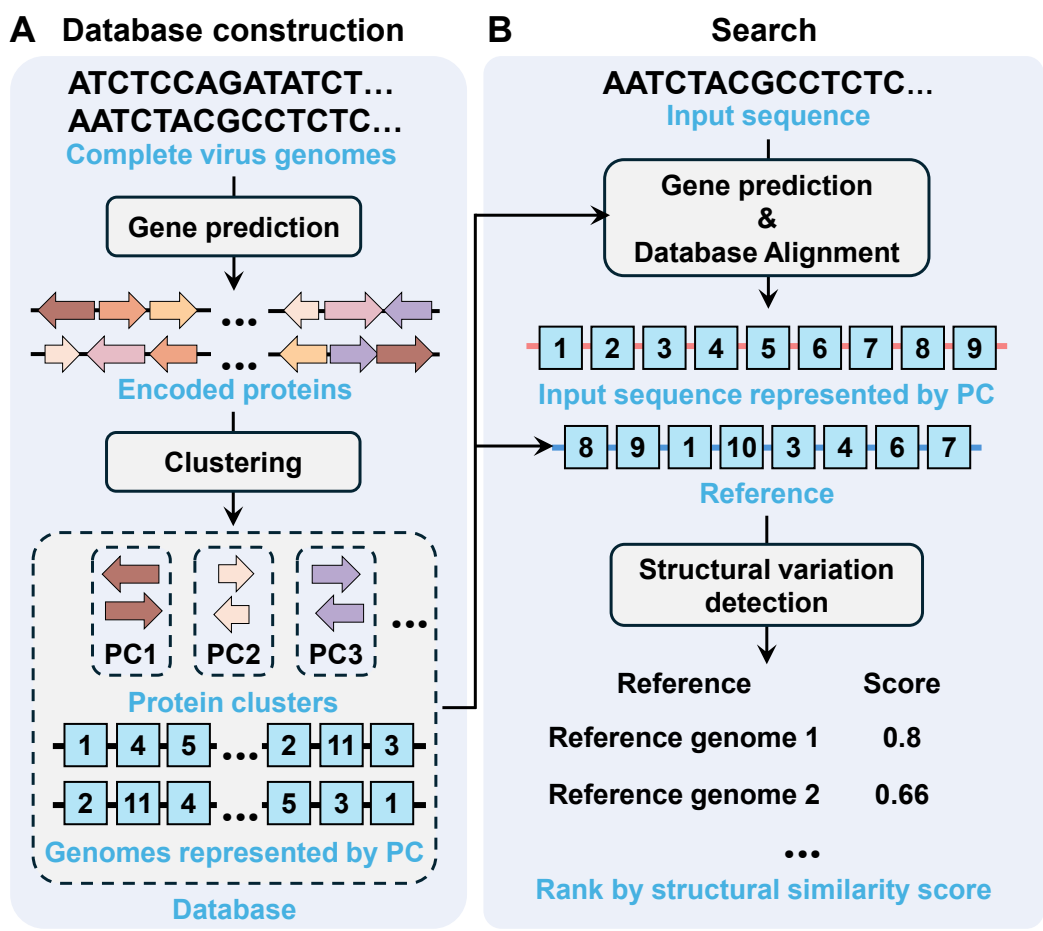

Fig. S1. The framework of the completeness estimation module (A) Construction of the complete genome and protein cluster database. Proteins encoded by complete genomes are clustered into protein clusters. Each genome is represented by a series of protein clusters. (B) Homology search for the most related reference based on protein organization. Given an input sequence, the encoded proteins are predicted and aligned to the database. Then the input sequence is converted to a protein cluster sequence. Structural variations (SVs) in the sequence are detected and the protein organization similarity score is calculated. The references are ranked based on the protein organization similarity.

1,651 assemblies. We further used BLASTN to align these microbial genomes against the viral sequences and removed any identified proviral regions, ensuring that the negative samples contained only microbial sequences.

To address the need for contamination detection in contigs, we fragmented the genomes into contigs of varying lengths. For each genome, we repeatedly selected random start positions and extracted fragments of 200-3000 bp. These fragmented contigs were used as the training set for the DNA branch of the contamination detection module.

For the protein branch, proteins in the training set were predicted using Prodigal-gv. All predicted proteins were clustered using MMseq2 with an identity threshold of $>50\%$ and coverage $>80\%$. Protein clusters with a purely viral origin and those with a purely cellular origin were used as positive and negative training data, respectively. Protein clusters shared by both origins were excluded from model training to avoid introducing ambiguity into the model.

Provirus dataset To evaluate contamination detection, we downloaded a set of provirus sequences from a previous study (Casjens, 2003). This dataset contains 50 bacterial genomes spanning 30 genera with 241 manually annotated proviruses. Because viral contigs obtained from sequencing and assembly are often fragmented and lack complete proteins at their ends, we simulate contigs with various levels of contamination percentage while preserving realistic host-virus junctions. We extracted each viral region along with its flanking host regions. We then segmented them into viral contigs contaminated with bacterial regions at predefined percentages (10%, 20%, and 50%) and with target lengths of 5, 10, 20, and 50 kbp. As controls, we also generated contigs with no bacterial region and purely bacterial contigs from the provirus sequences using similar procedures.

Leave-one-taxon-out dataset For the completeness estimation module, we constructed benchmark datasets using a leave-one-taxon-out strategy. Viral genomes were downloaded from the NCBI RefSeq database (release 218). Specifically, we retained complete viral genomes by filtering for sequences that are annotated as “complete”. The resulting dataset comprised 13,715 assemblies and 17,007 viral genomes from 2,423 genera, representing a wide diversity of viral species. For genera containing more than one

Table S1. Summary of the datasets used in the experiments.

Dataset	Description
	Contamination detection
Training dataset	The positive data includes 49,929 viral genomes downloaded from the NCBI RefSeq database and a set of high-quality metagenomic viral sequences from previous studies (Guo *et al.*, 2021). The negative data includes 1,651 bacterial and archaeal assemblies downloaded from the NCBI RefSeq database. To train the DNA branch, all sequences were fragmented into contigs of varying lengths. To train the protein branch, proteins predicted by Prodigal-gv were clustered using MMseq2 with an identity threshold of $>50\%$ and coverage $>80\%$. Protein clusters with a purely viral origin and those with a purely cellular origin were used as positive and negative training data, respectively.
Test dataset	A total of 241 provirus sequences that were manually identified from 50 bacterial genomes across 30 distinct genera (Casjens, 2003). These genomes were cut into contigs that contain different percentages of bacterial regions (0%, 10%, 20%, 50%, 100%) with varying lengths (5, 10, 20, and 50 kbp). Genomes from the same genus as test sequences are removed from the training set.
	Completeness estimation
Leave-one-taxon-out dataset	17,007 complete viral genomes from 2,423 genera were downloaded from the NCBI RefSeq database. For genera containing more than one species, we randomly distribute the species to reference and test sets in a ratio of nine to one. Then, we applied the same strategy at the genus and family level, resulting in three separate datasets with increasing difficulty. Sequences were randomly fragmented to create contigs with completeness levels ranging from 20% to 100%.
	Metagenomic sequencing data
Jinghu dataset	A total of 84,745 contigs longer than 10 kbp from surficial water of Jinghu Lake (China), a lake that is replenished by reclaimed water (Wang *et al.*, 2024).

species, we randomly distribute the species to reference and test sets in a ratio of nine to one. This leave-species-out strategy ensures a balanced distribution of viral taxa while ensuring that there is no overlap between the reference and test sets. Then, we applied the same strategy at the genus and family level, resulting in three separate datasets with increasing difficulty. To simulate realistic scenarios, complete genomes in these datasets were randomly fragmented to create contigs with completeness levels ranging from 20% to 100%.

Jinghu dataset We retrieved water metagenome data of 30 surficial water samples from Jinghu Lake, Tianjin, China (Wang *et al.*, 2024). The lake is recharged with reclaimed water, treated wastewater that is processed and purified by wastewater treatment plants (WWTPs). 84,745 contigs that are longer than 10 kbp were downloaded for analysis.

1.4 Metrics

We adopt the absolute error E_{cont} to evaluate the performance of ViralQC in contamination detection. Each base in an input contig belongs to either a viral or non-viral (i.e., contamination) region. Thus, E_{cont} is the percentage of misclassified bases (i.e., n) in a contig of length l, as shown below.

$$E_{cont} = \frac{n}{l} \quad (1)$$

In addition, we calculate the false positive rate (FPR) to assess the proportion of pure viral contigs incorrectly classified as contaminated. The FPR is defined as the proportion of pure viral contigs that are incorrectly classified as contaminated, calculated across all pure viral contigs in the dataset. The formula is shown below, where FP and TN represent the number of false positives and true negatives, respectively.

$$\text{FPR} = \frac{\text{FP}}{\text{FP} + \text{TN}} \quad (2)$$

With regard to the completeness estimation, we calculate the error E_{comp} between the estimated completeness $\hat{y}$ and the true completeness y. We also employ the prediction rate (PR) which is the proportion of contigs with prediction across all contigs:

$$E_{comp} = \hat{y} - y \quad (3)$$

$$\text{PR} = \frac{\text{Number of contigs with prediction}}{\text{Number of contigs}} \quad (4)$$

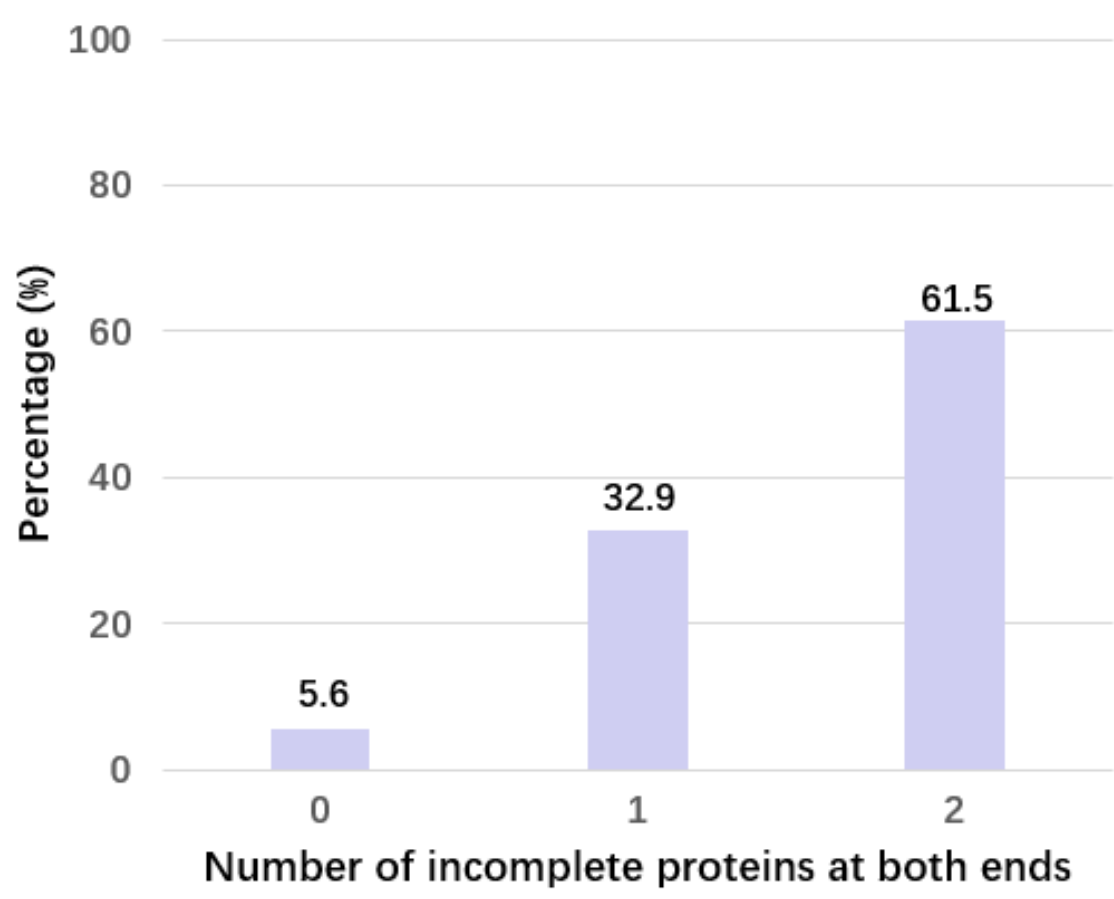

Fig. S2. The number of incomplete proteins at identified viral contigs' ends. Y-axis: the percentage of contigs that have 0/1/2 incomplete proteins at their ends. A total of 13,446 viral contigs are predicted by both geNomad and VirSorter2 in the lake metagenome as described in Supplementary Section 2.2. Among these contigs, more than 60% contigs do not contain complete proteins at both ends due to the limitations of sequencing and assembly.

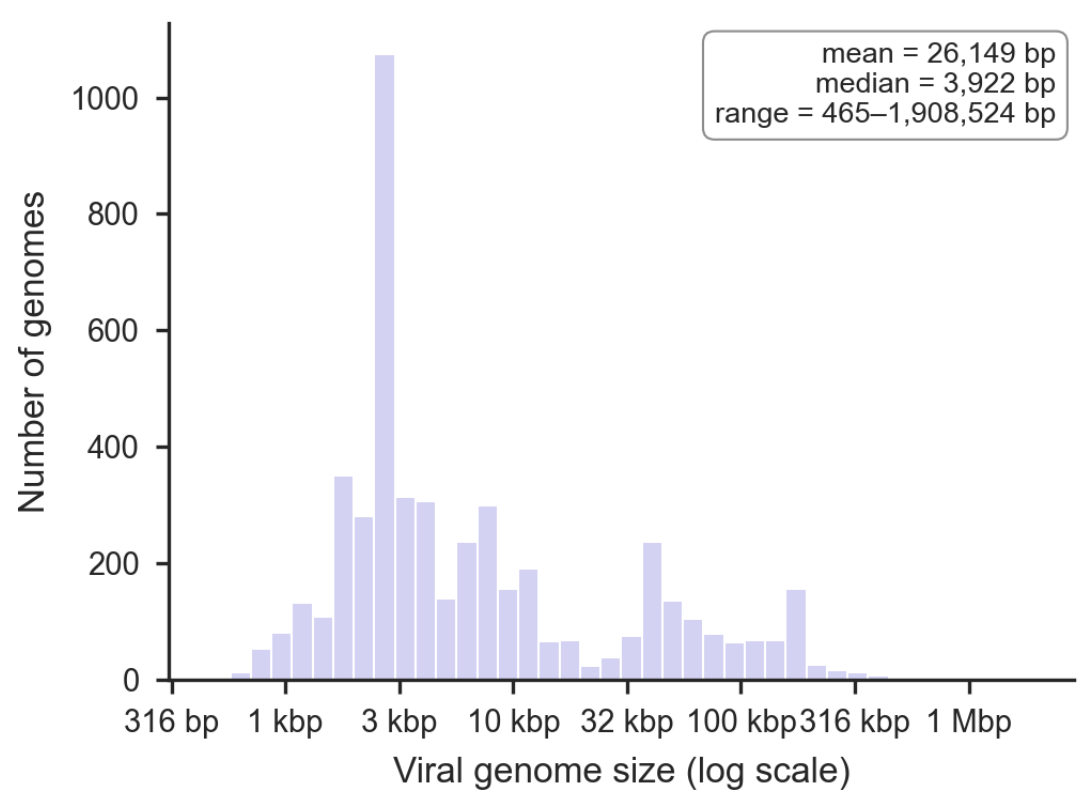

Fig. S3. Size distribution of viral genomes in the leave-one-taxon-out dataset. Histogram showing the genome-size distribution (log-scaled x-axis) of the viral genomes used in the leave-one-taxon dataset. Genome sizes span a broad range (465 bp – 1.91 Mbp; mean = 26,149 bp; median = 3,922 bp), with approximately 56% of genomes shorter than 5 kbp.

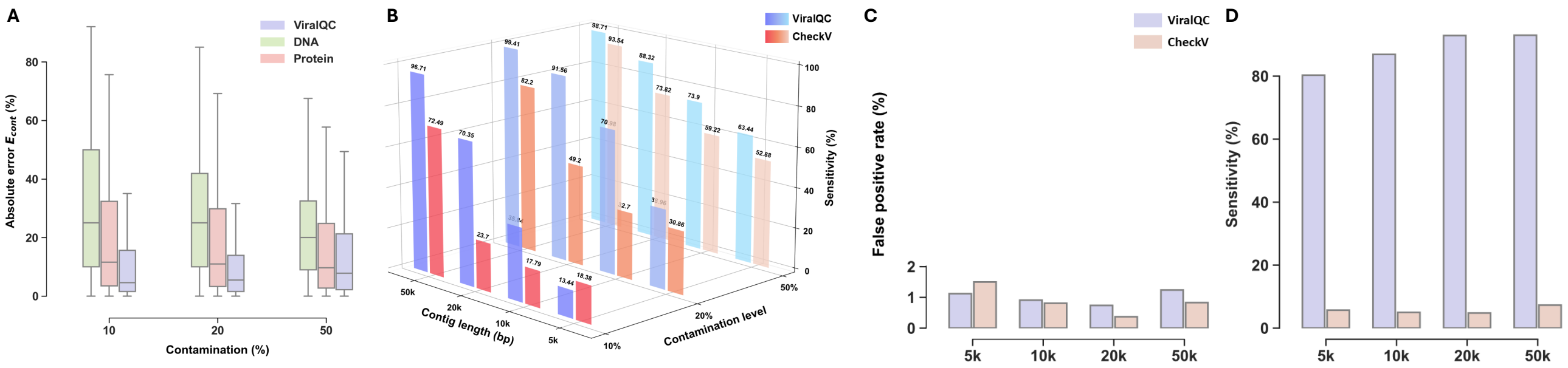

Fig. S4. (A) Comparison of ViralQC's contamination detection performance using only DNA or protein features on the provirus dataset. X-axis: different contamination levels (10%-50%). Y-axis: the distribution of absolute error E_{cont}. The model utilizing protein features outperforms the one using DNA features. This is likely because protein sequences, composed of a larger alphabet (25 amino acids), provide richer information compared to DNA sequences. While the model using protein features alone performs well, combining both DNA and protein features significantly enhances performance, emphasizing the importance of multi-modal feature integration. (B) Percentage of identified provirus sequences on contigs with varying lengths (5-50 kbp) and contamination levels (10%-50%). Cool colors present ViralQC and warm colors correspond to CheckV. As all the inputs contain contamination, the Z-axis represents the proportion of provirus sequences detected by each tool (sensitivity), where higher bars represent better performance. Across all lengths and contamination levels, ViralQC detected 86% of proviruses, compared with 62% for CheckV. (C) Percentage of misidentified provirus sequences on contigs of purely viral origin. Y-axis presents the false positive rate, which is around 1% for ViralQC. (D) Percentage of pure bacterial sequences identified by tools.

2 Supplementary for Results

2.1 Completeness estimation

2.1.1 Benchmark of bin mode

We compared the single-contig mode against the bin mode, where multiple contigs are jointly treated as a single genomic unit. We selected complete viral genomes encoding at least 15 proteins from the leave-one-taxon-out dataset. Each genome was partitioned into consecutive 5-gene fragments, which were then shuffled and provided to ViralQC as a single bin, and the resulting completeness estimates were compared against those produced by single-contig mode on the same genomes (Table S2). Across all three taxonomic levels, bin mode incurred only a marginal increase in mean absolute error: +0.01% at species, +0.15% at genus, and +0.42% at family level. These results indicate that, despite the absence of gene-order information, bin mode still provides accurate estimates and constitutes a useful extension for assessing viral bins recovered from metagenomic binning pipelines.

Leave-one-taxon-out	Single-contig mode	Bin mode	Δ(bin-single)
Species	1.29	1.30	+0.01
Genus	2.31	2.45	+0.15
Family	28.46	28.88	+0.42

Table S2. Average absolute error (%) of completeness estimation under single-contig mode and bin mode, evaluated on complete viral genomes (≥ 15 proteins) partitioned into 5-gene fragments. Results are reported under three leave-one-taxon-out settings (species, genus, family). Lower values indicate better performance.

2.2 Application to a water metagenome

We analyzed the water metagenome from Jinghu Lake, a lake that is replenished with reclaimed water. While reclaimed water helps mitigate water scarcity, the presence of viruses in the water raises significant concerns about its safe reuse and potential risks to human health. Thus, in this experiment, we applied ViralQC to assess the quality of predicted viral contigs in the Jinghu Lake samples. A brief description of the experiment is shown in Fig. S5. In addition, we examined whether quality examination of viral contigs can lead to better virus characterization such as taxonomic classification. To compare the performance on real data, we also ran CheckV for contamination detection and completeness estimation.

First, viral candidates were called with VirSorter2 (Guo *et al.*, 2021) and geNomad (Camargo *et al.*, 2024), identifying 14,779 and 14,205 viral contigs, respectively. Of these, 13,446 contigs were identified by both tools, while 1,333 and 759 contigs were unique to VirSorter2 and geNomad.

All viral candidates were then analyzed by ViralQC and CheckV to detect non-viral regions. ViralQC flagged contamination in 688 contigs, and CheckV in 527. We compared results based on the identification tool (VirSorter2-unique, geNomad-unique, and shared) in Fig. S6 A. On unique contigs, ViralQC identifies 18% more contaminated contigs than CheckV. More than 70% of these contigs were estimated to contain over 50% contamination, and over 40% exceeded 90%, suggesting many are false positives carried from virus identification tools. Protein annotations of detected contamination regions against the NR database show that 257 out of 270 regions contain only bacterial proteins, further supporting the ViralQC's results. Notably, contigs unique to geNomad had less contamination than those unique to VirSorter2 (8% vs. 24%), suggesting geNomad yields cleaner viral predictions. Among contigs called by both tools, only 385 (2%) were contaminated, underscoring the importance of using multiple tools for reliable virus identification. To minimize false positives, we therefore focused subsequent analyses on the 13,446 contigs shared by VirSorter2 and geNomad.

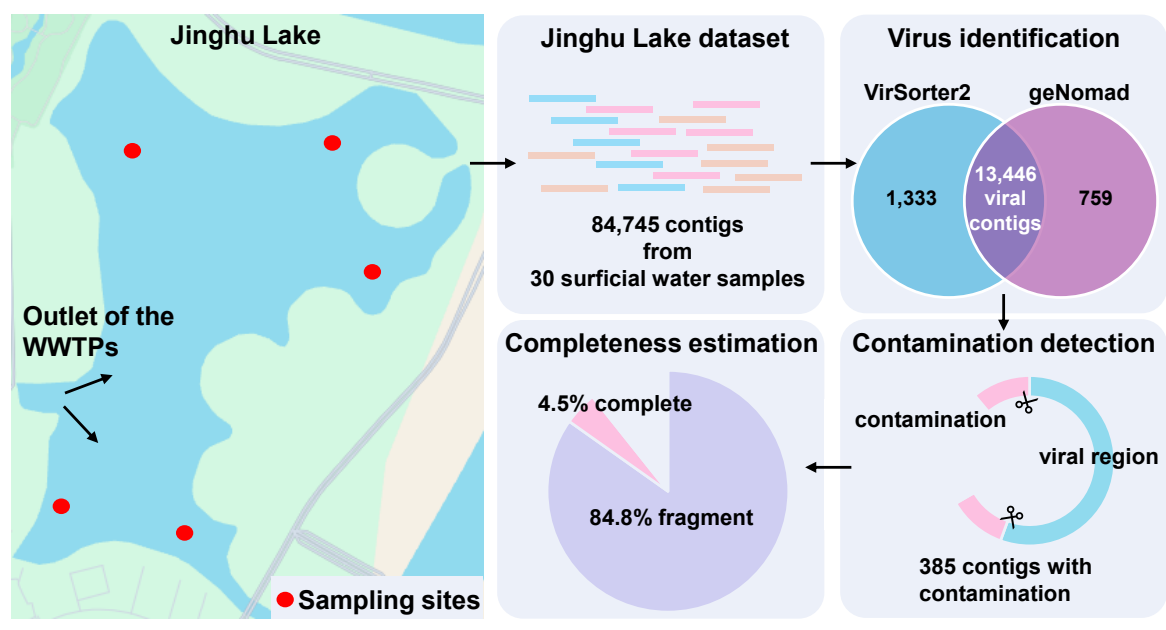

Fig. S5. A brief description of the sampling site and analysis pipeline. The Jinghu Lake dataset was collected from five sampling locations within the lake. Viral contigs were identified from the dataset using VirSorter2 and geNomad. Then, ViralQC was applied to the identified viral contigs for contamination detection and completeness estimation.

Since contamination can jeopardize the accuracy of taxonomic classification, we investigated whether the taxonomic classification

would change after the contamination removal. We used CAT v6.0 (Von Meijenfeldt *et al.*, 2019) with the NCBI virus database to classify the 385 contaminated contigs from the shared set. After contamination removal by ViralQC, 7% of the contigs were assigned to different taxa. For example, contig_1860139 was reassigned from *Nucleocytoviricota* to *Uroviricota*. These findings demonstrate that contamination can mislead downstream analyses such as taxonomic classification, particularly in complex environments where microbial and viral sequences are heavily interwoven.

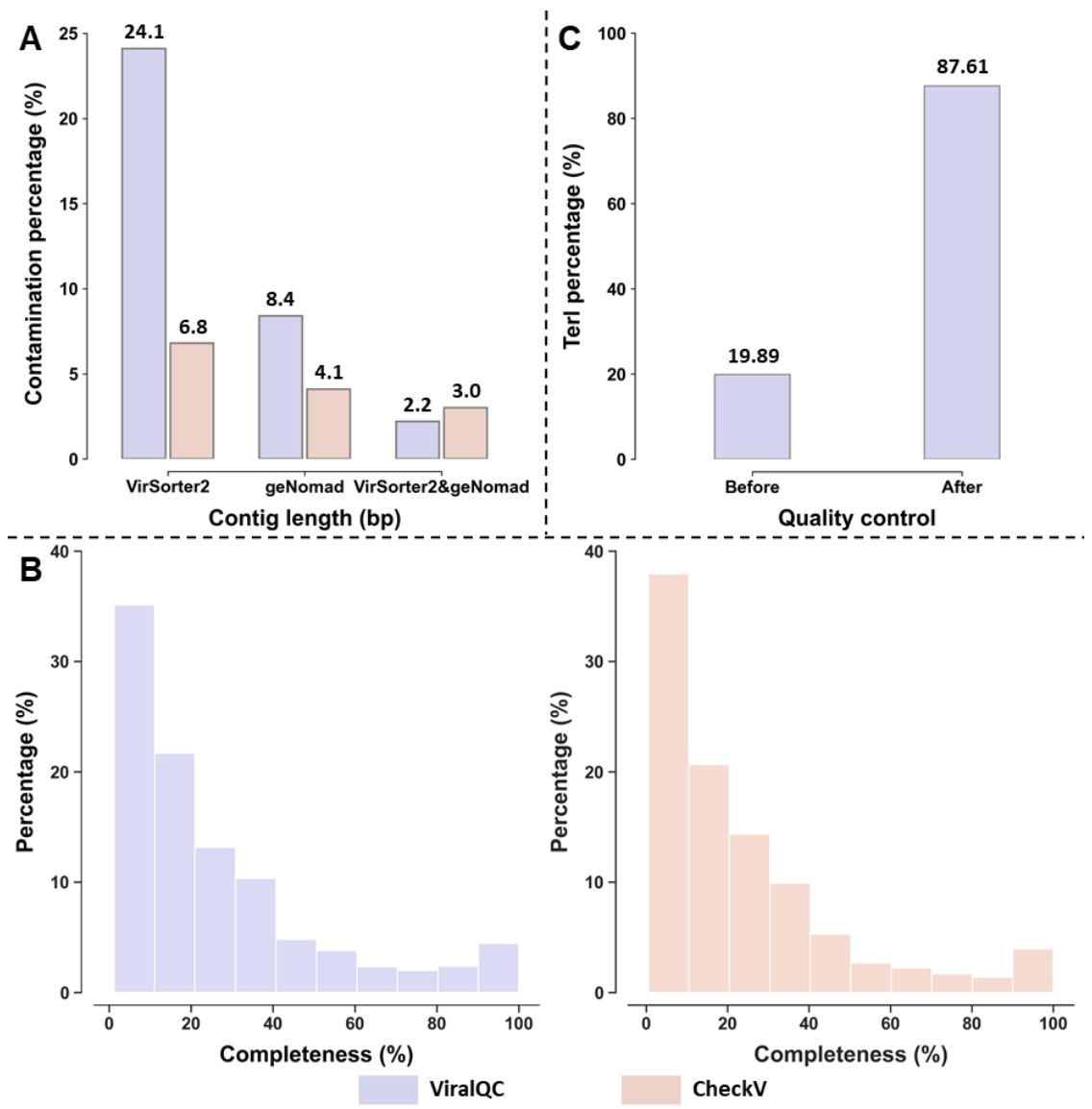

Fig. S6. (A) The percentage of contaminated contigs in VirSorter2-unique, geNomad-unique, and shared contigs identified by ViralQC and CheckV. (B) The distribution of high-confidence completeness results on decontaminated contigs estimated by ViralQC and CheckV. X-axis: estimated completeness. Y-axis: the percentage of contigs. (C) The comparison of the percentage of identified terminase large subunits in all contigs and near-complete contigs ($>$90% completeness). X-axis: before and after filtering near-complete contigs. Y-axis: the percentage of identified TerLs.

After removing non-viral regions from the contaminated contigs, we assessed the completeness of the viral contigs using ViralQC and CheckV. ViralQC produced high-confidence estimates for 2,586 contigs, and CheckV for 3,547 contigs. The distribution of high-confidence completeness is displayed in Fig. S6 B. Overall, the results from CheckV display a similar distribution to ViralQC. Among the contigs, 84.8% were genome fragments (completeness <50%). In contrast, only 4.5% were classified as complete or near-complete sequences (completeness $\geq$90%), with genome lengths ranging from 10 kbp to 200 kbp. The results suggest that the majority of viral sequences in the Jinghu Lake metagenome are fragmented, likely due to the inherent complexity of the water microbiome and the challenges associated with sequencing and assembling. Furthermore, the low proportion of complete/near-complete viral genomes highlights the need for additional refinement or complementary tools to improve genome recovery.

We further analyzed the contigs classified as prokaryotic viruses (*Caudoviricetes*), which constitute 85% of all viral contigs. PhaBOX2 (Shang *et al.*, 2023) was applied to identify terminase large subunits (TerLs) encoded in the sequences, which are among the most widely distributed marker genes. It is estimated that TerLs appear in over 97% of prokaryotic viruses. However, TerLs were detected in only 19.89% of all contigs, compared to 87.61% in complete or near-complete contigs estimated by ViralQC (Fig. S6 C). While the true lengths of their complete genomes are unknown, this difference offers additional evidence supporting the reliability of ViralQC's completeness estimation.

2.3 Time and memory

We measured the running time and peak memory usage during execution of ViralQC and CheckV on 5,000 contigs of length 10 kbp. They were run on the Intel® Xeon® Gold 6258R CPU with 8 cores and a single NVIDIA GeForce RTX 4080 GPU. We also record the disk storage required for installation (primarily databases and models). As shown in Supplementary Table S3, ViralQC takes more time and memory to make the prediction due to its use of large language models, but it requires less disk space to set up the database.

Program	ViralQC	CheckV
Elapsed time (min)	63	33
Peak memory (GB)	5.89	2.66
Disk size (MB)	447	6400

Table S3. The elapsed time, memory usage during execution, and the required disk space of ViralQC and CheckV.

References

Camargo, A. P., Roux, S., Schulz, F., Babinski, M., Xu, Y., Hu, B., Chain, P. S., Nayfach, S., and Kyrpides, N. C. (2024). Identification of mobile genetic elements with genomad. *Nature Biotechnology*, **42**(8), 1303–1312.

Casjens, S. (2003). Prophages and bacterial genomics: what have we learned so far? *Molecular microbiology*, **49**(2), 277–300.

Fernández-Pato, A., Sinha, T., Garmaeva, S., Gulyaeva, A., Kuzub, N., Roux, S., Fu, J., Kurilshikov, A., and Zhernakova, A. (2025). Early-life development of the gut virome and plasmidome: A longitudinal study in cesarean-born infants. *Cell Reports*, **44**(6).

Guo, J., Bolduc, B., Zayed, A. A., Varsani, A., Dominguez-Huerta, G., Delmont, T. O., Pratama, A. A., Gazitúa, M. C., Vik, D., Sullivan, M. B., *et al.* (2021). VirSorter2: a multi-classifier, expert-guided approach to detect diverse DNA and RNA viruses. *Microbiome*, **9**, 1–13.

Merrill, B. D., Ward, A. T., Grose, J. H., and Hope, S. (2016). Software-based analysis of bacteriophage genomes, physical ends, and packaging strategies. *BMC genomics*, **17**(1), 679.

Shang, J., Peng, C., Liao, H., Tang, X., and Sun, Y. (2023). Phabox: a web server for identifying and characterizing phage contigs in metagenomic data. *Bioinformatics advances*, **3**(1), vbad101.

Tisza, M. J., Pastrana, D. V., Welch, N. L., Stewart, B., Peretti, A., Starrett, G. J., Pang, Y.-Y. S., Krishnamurthy, S. R., Pesavento, P. A., McDermott, D. H., *et al.* (2020). Discovery of several thousand highly diverse circular dna viruses. *elife*, **9**, e51971.

Von Meijenfeldt, F. B., Arkhipova, K., Cambuy, D. D., Coutinho, F. H., and Dutilh, B. E. (2019). Robust taxonomic classification of uncharted microbial sequences and bins with cat and bat. *Genome biology*, **20**, 1–14.

Wang, D., Shang, J., Lin, H., Liang, J., Wang, C., Sun, Y., Bai, Y., and Qu, J. (2024). Identifying arg-carrying bacteriophages in a lake replenished by reclaimed water using deep learning techniques. *Water Research*, **248**, 120859.
